# Disability, mental health, sexual orientation and gender identity: understanding health inequity through experience and difference

**DOI:** 10.1186/s12961-018-0366-1

**Published:** 2018-10-09

**Authors:** N. Nakkeeran, Barathi Nakkeeran

**Affiliations:** 1 Ambedkar University Delhi, Lothian Road, Kashmere Gate, New Delhi, 110006 India; 2 Independent researcher, New Delhi, India

**Keywords:** Health inequities in India, Health equity research, Disability, Mental health, Sexual orientation, Gender, AMCCON 2018

## Abstract

**Background:**

This paper focuses on inequities in health in the context of disability, mental health, sexual orientation and gender identity (The authors’ location outside of these identities is acknowledged as a serious limitation in discussing experience as a framework to understand health inequity in the dimensions of disability, mental health, sexual orientation and gender identity). These are dimensions that lead to health inequity primarily through the pathways of stigma and discrimination. The aim here is to distinguish the unique characteristics of these groups and thereby try and articulate a new understanding of health and health equity with identity and difference in the foreground. We aim to bring attention to experience as a crucial parameter to discuss health equity in this context.

**Discussion:**

Health inequity can be approached in two ways. One is to look at the lacuna in the current public health discourse in addressing the specific health concerns along the dimensions of disability, mental health, sexual orientation and gender identity. The second approach involves a more organic way of taking on board the concerns of these groups, rather than as after-thoughts; this involves a framework that gives a central role to the lived experience of stigma and discrimination.

The dimensions of disability, mental health, sexual orientation and gender identity affect health inequities constitutively, instrumentally through co-morbidities, and through stigma either directly or indirectly. Experience of stigma also forms the basis of identities and the difference between identities, which emerges as an important concept in the articulation of health inequities beyond measurable gaps. Recognition and representation of these differences then form the basis of an inclusive articulation on health.

**Conclusion:**

The centrality of difference and experience prompts us to problematise the idea of equity that rests on ‘avoidable and unfair’, ‘differentials’ and even to argue that such a definition based on differentials, used in a quantitative sense, seriously limits our understanding of health inequity. Health equity will therefore not merely mean ‘closing avoidable health gaps,’ but mandate an inclusive social arrangement that celebrates difference.

## Background

This paper is based on a set of three presentations that formed part of the session themed ‘Socially Constructed Vulnerabilities’ in the conference on Health Inequities in India: Transformational Research for Action in January 2018. The three presentations focus on health inequities among people living with a disability, by Renu Addlakha [1], health inequities among persons living with psycho-social disabilities and mental health conditions, by Bhargavi Davar [2], and health inequities among the Lesbian Gay Bisexual Transgender Queer Intersex (LGBT) communities, by Ketki Ranade [3]. These presentations brought out the complex ways in which disability, mental health, sexual orientation and gender identity redefine and reconstitute health inequity, enunciating many key arguments and concepts. Herein, we take forward and further elaborate on these concepts with the support of secondary literature.

Social science literature has acknowledged social class, caste and gender as structural determinants of inequalities and as viable dimensions (variables) for categorisation of groups. National level datasets, such as the National Family Health Survey [4] and the District Level Household and Facility Survey [5], use class, caste and gender as independent variables to see the distribution of differences in health and nutritional indicators. On the other hand, disability, mental health, sexual orientation and gender identity are generally not considered as categories to classify and compare groups but only to capture individual-level disparities. They do not assume the place of independent dimensions of analysis. The aim here is not to put these sets of groups against each other, but to explicate the nature of the differences between the two.

Disability, mental health, sexual orientation and gender identity are dimensions that lead to health inequities primarily through the pathway of stigma and discrimination, leading to exclusion from accessing resources needed to be healthy and health services through multiple pathways. The extent of the vulnerabilities stemming from these dimensions is, to a great degree, mediated and configured by environment and society. Questions around identity and lack of recognition play a pre-eminent role in laying the ground for the material and distributional aspects of inequity [6].

While we try to underline the commonalities among these groups, they also have unique characteristics that distinguish them. The common and the unique aspects of these dimensions of vulnerabilities have the potential to challenge the popularly accepted understanding of health and health equity and offer scope to articulating a new understanding on health equity with a greater prominence to the ideas of recognition, identity and difference. While the three presentations on which we base this paper focused on the Indian scenario, for reasons of uneven availability of literature and not to miss out on any key theoretical arguments the paper depends on Indian as well as global literature.

The first section of the paper will look into the nature of the three groups, namely persons living with physical disabilities, with mental health conditions and LGBT^1^ groups, and examine their vulnerabilities and how they contribute to disparities in health individually as well as a constituent element of an intersecting set of sites/dimensions of vulnerabilities. The second and third sections of the paper will look at the concepts of health and equity in light of the nature of these dimensions of vulnerabilities.

## Health and health inequity along the dimensions of disability, mental health, sexual orientation and gender identity

### Disability and health inequities

Though disability has an unmistakable dominant physical basis, Addlakha [1], in her presentation, citing recent international conventions and guidelines [7–9], described disability as the “*interaction between persons with impairments, and attitudinal and environmental barriers that hinders their full and effective participation in society on an equal basis with others…*” [7]. She defined disability as the “*umbrella term for impairments, activity limitations, and participation restrictions*” [8]. Disability is conceptualised “*not solely as a problem that resides in the individual, but as a health experience, that occurs in a context*” [9]. Disability should not be treated simply as an outcome of a disease, trauma or other health conditions; therefore, it requires interventions to remove environmental and social barriers coupled with a shift in the societal attitude [10].

Once it is acknowledged that disability does not solely reside in the individual, one is automatically compelled to recognise the mediation of social, economic, locational and other privileges in shaping and determining the degree of disability experienced by specific individuals. For instance, in cities that have end-to-end disabled-friendly mobility arrangements, individuals having mobility-related physical impairment may experience disability very differently compared to those who live in cities that have not developed such infrastructure or those who cannot afford these facilities. Disabilities cannot, therefore, be clearly understood in a singular essentialist manner. Attitudinal and environmental barriers, as well as the intersectionality [11] of privilege and power, operate quite strongly to shape individuals’ experience of disability.

### Disability as a dynamic continuum

WHO estimates that more than a billion people live with some form of disability, which corresponds to approximately 15% of the world’s population. Underlying this significant statistic is the global understanding that disability is “*a continuum, relevant to the lives of all people to different degrees and at different times in their lives*”, virtually a “*universal phenomenon*” and a “*natural feature of the human condition*” [12]. The International Classification of Functioning, Disability, and Health strongly suggests for a shift from the notion that disability is a ‘minority’ problem to one that considers it as a universal human experience [13].

Facts call to question a common misconception that disabled persons are born with their disabilities. This misconception stems from and is entwined with a false dichotomy between ‘abled’ and ‘disabled.’ The dichotomy gets further challenged by the fact that disabilities vary in magnitude and occupy a continuum [8]. A disability could also be transient, as in the case of postpartum depression or blindness due to cataract; recognising and acknowledging this temporal aspect of disability within medicine and public health planning is important.

It is also important to recognise disability as a dynamically shifting continuum across the life-course. For instance, the gravity of disability that is largely acquired with ageing among the aged population may be trivialised by comparing it to congenital ones and those experienced throughout one’s life [14]. Either explicitly or implicitly, and in varying degrees, disability associated with ageing may be considered as unavoidable. In the context of a country like India, with relatively low life expectancy and competing demands on the health system from communicable and non-communicable diseases, such perceptions may influence health system attitudes to disabilities related to ageing.

Before we close this brief discussion, it is important to emphasise that, notwithstanding the socially mediated experience of disability, there is a need to retain the biological basis of disability within this discourse. While it is true that disabilities are socially mediated, the argument that it is solely socially mediated cannot be subscribed to, because doing so would dilute the specific health needs of disabled people and the special care they require [1].

From the literature, one is able to delineate four different attitudes and relational arrangements that get established in society with respect to persons with disability, namely eliminational, exclusionary, integrational and independent living. They are not conceived as sequential; in all probability, they co-exist or even surge past each other in different times and contexts, with changing values mediated by technological advancements and economic and social changes.

An eliminational attitude is an extreme attitude of getting rid of individuals once a disability is detected either during pregnancy or after birth. We have evidence of practices reflective of such attitude in the form of individual households deciding to do away with a child with a disability, with a medical doctor discarding children with disability in large numbers believing in political ideologies that promoted eugenics [15] or sections of populations preventing such births by terminating the pregnancies. The explosive growth in medical technology in recent decades has transformed this attitude into new-age eugenics and genetic enhancements [16]. These practices, therefore, range from occasional and individual to systematic and population wide, between esoteric and scientific as well as between out-right illegal to legally sanctioned.

An exclusionary attitude is the most common and naturalised attitude displayed by society towards people with a disability by excluding them either directly or indirectly, through social norms, institutional arrangements and insensitive design of the built environment, from accessing healthcare, education, employment and being full members of the society [17, 18]. Such exclusion can happen along a continuum ranging from protective paternalism to humiliating verbal or even physical abuse. This can get further complicated with other intersecting dimensions of vulnerabilities such as gender, caste and minority statuses [19, 20].

A protective-integrative attitude refers to the social accommodation of people with disability by being cognisant of and sensitive to their disability-related problems. Such integration aims at ensuring physical, economic and emotional security and usually entails changes in the family [21] and community perceptions and in institutional norms to ensure physical safety, provide a protective family space, facilitate higher longevity and, in some situations, enable an economically active life.

Independent living as a value takes the expectations to the highest level, wherein the society provides an attitudinal, social, physical and policy environment that facilitates people with disability to lead an independent life as a full member of the society on their own terms [22].

These diverse attitudinal and relational arrangements and response from society have relevance to health as these values also shape how the health system and associated policies view disability and respond to it. These values will imbue meaning to health, wellbeing and avoidable or unfair differentials, and may also determine the value system that underlies healthcare systems. For instance, societies that foster a value of disabled people’s right to lead an independent life may subscribe to an understanding of health that recognises the perspectives about the health of people with disability.

### Disability, health and health inequity

The debate surrounding health inequity of the disabled is uniquely situated because, even though disability is about body and physicality and has direct linkages with medicine, the disabled are marginalised with respect to health. People with disability navigate and experience health systems differently, and this experience itself constitutes health inequity. However, this is not captured when inequity is explained through differentials. Since the health system is not designed to keep in mind the requirements of people with disability, disabled people have to negotiate and challenge the norms and structures of the health system during the course of every encounter. The needs of disabled people are incorporated into health systems as after-thoughts rather than as “*universally designed*” [8] systems with a seamless experience for disabled people. Inclusiveness has to be built into the entire spectrum of services and experience ranging from, for example, accessing the toilets [23], to attitudes, interactions and language of service providers. Therefore, for a health system to be inclusive, it must go beyond being merely non-discriminatory to being more actively engaging, affirmative and promoting of a disabled-friendly environment. Beyond the environment, inclusivity must also be about the language used and attitudes. For instance, ramps should be part of the original design of any public building. However, many buildings, including public places, often disregard such basic “*accessibility details*” [24], thereby either discouraging people with disability from accessing these facilities or reinforcing their dependency on others.

In addition to this constitutive role, the response of the health system towards people with disability can also affect their perception of the health system and play an indirect role in influencing the ease and extent to which disabled people access the healthcare system, apart from affecting their self-esteem and making them feel stigmatised. Discrimination in the form of rude behaviour, verbal abuse, bullying, delay in providing treatment, not respecting their privacy and dignity, negligence extending as far as physical violence and even sexual exploitation of disabled people are not infrequent in the health system context. As a result of such discrimination, people with disabilities feel further inhibited to access even essential health services, leading to non-treatment or delayed treatment.

Studies show that the prevalence of certain health conditions such as obesity, smoking, hypertension and diabetes is higher among persons with disabilities [10]. Even the severity of an ailment or disease has been found to be relatively greater among people with disabilities. Further, disabled people are more prone to injuries and accidents, the proportion who are able to engage in regular physical activity and receive preventive care is smaller, and treatment outcome and disease management are poor. Another frequently reported problem has been the exclusion of people with disability from medical insurance coverage [1, 23] or being asked to contribute higher premiums. While it is convenient to attribute these issues as unavoidable outcomes of disability, a robust and fair health system has to account for and respond to these requirements at the very least. What is fair and unfair or what is avoidable and unavoidable is sometimes adjudged differently by society and health systems, depending upon the underlying values. For instance, in countries that emphasise independent living, the range of facilities that are made available and accessible are vastly different from those in other countries, where even the most essential facilities may be considered as not basic [23]. The stigma associated with disability further complicates the situation with heightened stress, which could in turn further affect the health status of people with disabilities.

Persons with disabilities are at a higher risk of being discriminated against in accessing education, remunerative employment opportunities and other welfare services, thereby affecting their livelihood and quality of life, and thus, indirectly, their health status. Further, a greater proportion of adults with disability stay unmarried and lack family support, therefore becoming deprived of social interaction. Studies also report that the disabled are more vulnerable during disasters [22].

Equity should be visualised not only as helping persons living with disabilities to become successful beneficiaries or consumers of the health system but also as providing opportunities for and enabling persons with disabilities to become healthcare providers and key decision-makers in the health system [25].

### Issues around measurement and data

Assessing the magnitude of disability across a meaningful classification is important to provide visibility to the concerns of people with a disability, which in turn will provide scope for informed and sensitive policy-making. Such an assessment by a state agency provides legitimacy to the problems in terms of recognising the magnitude as well as to the vocabulary and nomenclature.

For instance, the Indian Census 1981classified the disabled into three problematic categories, namely “*dumb*”, “*crippled*” and “*blind*” [14], reflecting the charity-based discourse on disability that prevailed during that time. By 2011, the categories were refined into more composite functional categories such as seeing, hearing, speech, movement, mental retardation and mental illness [14]. The Rights of People’s with Disability Act, 2016, increased the types of disabilities recognised from 7 to 21 [14]. However, an exercise of assessment should not stop merely at adding many categories or counting the number of disabled people, but should include efforts to provide a numerical representation of the disabled population as a constituent of national demography similar to age, caste and gender.

Arriving at an accurate estimate of the number of persons living with a disability is difficult because of reasons such as the absence of a universally accepted definition of disability and the dynamic nature of disability across the lifespan. However, focusing on gathering accurate data poses the danger of entering a regressive path with respect to understanding and analysing disability.

### Mental health and health inequity

Davar presented her experience with an urban community mental health and inclusion programme [2]. She underlined that the current international understanding of community mental health programmes emphasises not merely enabling persons to be symptom free but their inclusion into the mainstream as full members of the society. As a corollary, mental health services should be community based and integrational and not focused on institutionalisation that isolates the individuals from the society and excludes them from accessing any welfare services. Her presentation also highlighted the need to acknowledge and address the role of structural factors, such as poverty, lack of civic amenities, violence, hunger and starvation, lack of livelihood opportunities and migration, when one works on mental health.

Despite its huge contribution towards burden of disease [26] and its strong interrelationship with other public health issues [27] and inequities [28–32], mental health concerns are perhaps the most neglected aspect of public health worldwide [28, 30, 33]. In any given year, globally, approximately 30% of the population is affected by mental disorders, of which over two-thirds remain untreated or under-treated. Approximately 14% of the global disease burden is from common mental disorders, and this proportion is only likely to increase in the coming decades [30].

Under-recognition of mental health concerns is a problem at all levels, from the international policy arena and national health system level to community and household levels. Most national health systems display their low priority to mental health concerns through low financial investments and outlay [28, 34], poor human resource presence [33–35], shortage of supplies meant specifically for managing mental illnesses [28], and institutional level discrimination of individuals with mental illnesses. At the community and household levels, there is a wide prevalence of stigma and secrecy, on the one hand, and non-recognition and non-treatment of mental illnesses on the other [36].

Mental health is the principal pathway through which environmental and social stressors get embodied in individuals’ lives. For example, the effects of rapid economic changes on economic status and livelihood; of conflict, violence and displacement, of discrimination stemming from social location/caste, ethnicity, disability, gender and sexual orientation, or of individual stressors associated with work, family, marriage, social norms and expectations, get inscribed onto individuals’ lives and are expressed as enduring patterns of behaviour or as health concerns. In other words, the domain of mental health has the potential to simultaneously play an embodying and constituent role in an intersectional context. This means that mental health concerns are more prevalent and pronounced when coupled with other dimensions of vulnerabilities and discrimination stemming from poverty [2, 31], caste, race, gender and sexual identities [3, 33, 37], and disability.

Poor mental health status has been found to contribute towards inequity in general health status. People with mental illness reported co-morbidities, including malnutrition and severe anaemia, high rates of not opting for treatment, delayed treatment, high rates of non-adherence to treatment, and low rates of hospitalisation. Additionally, they experienced a higher mortality rate from conditions such as ischaemic heart diseases and cancer [2, 27, 28, 38]. There was also a preponderance of high-risk behaviour and substance abuse [39] among persons living with mental health conditions, and public health campaigns had little effect on them compared to others without mental disorders [28]. Individuals with mental illness are often required to pay higher insurance premiums for the same physical conditions than those without such a history. Further, the role of mental illnesses in contributing to health inequity may also be inter-generational; for example, maternal depression after childbirth has been found to be an independent risk factor for a child’s low birth weight, malnutrition, growth faltering and incomplete immunisation [33, 40].

Availing care for mental illness is a serious challenge due to lack of awareness, stigma, an abusive healthcare environment, lack of appropriate care and trained human resources at primary care level, and high healthcare costs [41]. This is especially severe for those with economic vulnerability. Davar observed [2], based on her field experiences, that among an urban poor population, people with a mental disorder like depression and anxiety needed, as the first line of treatment, comprehensive healthcare that would also address malnutrition. However, it was often difficult to negotiate with healthcare providers to extend primary healthcare services to these individuals experiencing mental health problems. On the other hand, those who provided mental health services were separate groups of practitioners who were not in a position to provide general health services. Moreover, urban-poor settlements had no services for addressing alcoholism and de-addiction.

People with severe mental health issues have a high risk of being abandoned by their family, often resulting in them being involuntarily institutionalised. Institutionalisation generally reduces access to welfare services, healthcare, proper nutrition and family support, and many of these individuals have high rates of mortality [2]. Involuntary admission into mental hospitals is still a widespread practice across the globe, amounting to over one-tenth of all admissions, and this proportion is far higher in certain kinds of facilities and certain regions [34]. Recent policy efforts discourage institutionalisation and promote inclusion in society. International and national policies aim to go beyond providing mental health support and reducing the burden of disorder with the aim of encouraging people with mental illness to be able to live in the community as full and active members of society [7, 42–44].

Similar to what was discussed in the previous section on disability, here too the debate is between a medical model and a social model. The former defines mental illness as an individual condition that has to be addressed through medical care or public health interventions. In contrast, the social model looks at mental illness as a result of discriminatory structural factors imposed on individuals and groups [28, 30].

### Sexual orientation and gender identity as dimensions of health inequity

Ranade, in her presentation on health inequities among LGBT persons [3], highlighted that the assumption of heterosexuality and the gender binary of male and female that leads to the false categorisation of people as ‘normal’ and ‘abnormal’ is a fundamental problem ubiquitous in healthcare institutions, services, curricula and training. Further, Ketki also enumerated several situations of discrimination faced by the LGBT community within the health sector.

In the context of sexual orientation and gender identity, health inequity operates primarily through stigma and discrimination. “*A general starting point … to fight discrimination … is a clearer understanding of how people create and manifest their sexual practices, and if and how this is part or not part of forming identities*” [45]. Sexual orientation and gender identity may be understood as two different yet interrelated dimensions of an individual’s identity. Sexual orientation refers to “*a person’s sexual and emotional attraction to another person*” and the associated behaviour and social affiliation [46]. In other words, it is not to be identified with the mere physical act of sexual intercourse, but it should also include the aspects of attraction for and the identification as lesbian, gay, asexual or bisexual, among others [47]. Gender identity, on the other hand, refers to a person’s deeply felt and individual experiences of gender [48]. Despite the mainstream understanding that gender resides in individuals, the important role of societal structure, cultural expectations and personal interactions in its development has also been recognised. For instance, Butler would argue that there is no “*natural body that pre-exists its cultural inscription*” [49]. Often, to denote a “*sexual orientation or gender identity that does not conform to dominant social norms*” [50] the term ‘queer’ is adopted by individuals. Many may also adopt ‘queer’ as an identity term “*to avoid limiting themselves to the gender binaries of male and female or to the perceived restrictions imposed by lesbian, gay and bisexual sexual orientations*” [50]. There are other queer identities, like *hijra* and *kothi* in India, which are indigenous identities that are often mapped onto western terms but do not completely overlap because of the cultural specificity of these identities. However, trans persons may decline the use of such terms to identify themselves and some may find such terms derogatory. Many have therefore preferred the usage of the term ‘trans’ in place of these indigenous identities. We acknowledge that it is best left to individuals themselves to choose the terms to be used to define their identity, based on what they find most comfort and power in.

### Role of stigma, social exclusion and discrimination in shaping inequities for LGBT individuals

There is a tendency to hyper-sexualise the LGBT groups’ identity, resulting in LGBT groups’ being wrongly labelled as sexual offenders. For a long time, homosexuality was viewed as pathological and used to be classified as sexual deviance [3]. In India, homosexuality is criminalised, vide the archaic Section 377 of the Indian Penal Code [51], and is still most often improperly grouped with deviant behaviour [3, 45]. As of July 2018, a plea seeking the decriminalisation of Section 377 is under judicial process. Though the recent judicial trend indicates a possible positive outcome, it would be premature to comment on the verdict of the Supreme Court [52]. LGBT individuals are identified solely on the basis of their sexual orientation and gender identity, whilst in the process subjecting them to humiliation and indignity. At the same time, the cis-genderist and heteronormative population’s refusal to accept LGBT individuals as regular members of society pushes the LGBT community to its margins either physically or in terms of social visibility. Hence, they face the “*conundrum of being both hypervisible and invisible*” [53]. As a result of such a stigmatised status, LGBT individuals face various forms of violence, bullying and repression. Often, law enforcement agencies themselves target sexual minorities and gender-expansive individuals, perpetrating rape and other forms of sexual violence [37, 54–60]. Even within health systems, they are severely discriminated against. For instance, registration forms at hospitals have columns that only provide for male and female. Further, trans-women, for instance, are often admitted in male wards despite them expressing their gender [3]. Gender dysphoria is not taken seriously by many medical professionals and, when trans people approach endocrinologists or surgeons, they are often told that gender reassignment treatments are unnatural or unethical [3].

Families often reject LGBT individuals [56]. Neighbourhoods are suspicious of individuals not conforming to their idea of ‘normal’, thus subjecting LGBT individuals to violence and humiliation. Stemming from the notion that non-heteronormative is unnatural and sinful, religious groups target and attack LGBT persons [57]. An appalling number of instances of trans people being persecuted even to the point of death is witnessed across the world [58–60].

Pushed to a state of “*precarity*” [61], LGBT individuals (are compelled to) adopt a range of behaviour or collective norms to create safe spaces where they may be accepted in a manner and form they want to be, and have the freedom and choice to express their sexual orientation and gender identity without the fear of persecution. These behaviours can vary from suppressing or concealing a part or the whole of their identity, resorting to episodic escape from the mainstream or finding a long-term life outside their natal society [37]. Alternatively, individuals suppress or maintain the secrecy of their sexual orientation or gender identity. This feeling of suppression may be experienced differently by different individuals. Since the “*freedom to express sexual orientation and gender identity is closely connected to economic and political freedom*” [45], the ability to blend or to choose to be explicit and the consequent acceptance or rejection by mainstream society is again an operation of socioeconomic status [62]. For instance, one study in India “*found that 71% of Indian*” LGBT “*employees feel stalled in their careers, while only 57% of those not out feel stalled*” [47, 63]. In the context of denial of recognition, one wonders if health, defined narrowly, is the most central concern for LGBT individuals.

Apart from the direct effect of stigma on LGBT individuals’ health status, stigma also leads to their exclusion from education and employment, which in turn could affect their economic status, quality of life and, hence, health status. For instance, literature shows that “(L)*esbian, gay, bisexual and transgender (LGBT), and gender non-conforming* [sic] *student and staff, face bullying in educational institutions*” from both peers and teachers [64]. As a result of being discriminated against in workplaces and not having equal and fair access to jobs, many LGBT individuals take up jobs in less formal sectors. However, the LGBT community faces discrimination in almost all sectors, and therefore many of them are ultimately forced into sex work or begging. Another study done in India and Bangladesh among men who have sex with men (MSM), in 2005, found that 75% of respondents were engaged in sex work out of economic compulsion. Access to other economic activities were seriously restricted due to discrimination [47].

Discrimination may also operate through institutional structures and norms of health systems. Representation of LGBT communities in the health system at all levels is negligible, which adds to a lack of awareness and sensitivity towards the needs of the LGBT community. As a result, even institutions, processes, infrastructure, technology and norms of delivering healthcare are insensitively structured. A 2011 study in Kenya interviewed 474 respondents about discrimination and found evidence of discrimination by healthcare providers as one form of discrimination and harassment [65]. The very design of a health system or any service provided is orchestrated for the needs of the mainstream, rarely considering the needs of the individuals who do not fit into the mainstream definitions. Individuals in the so-called ‘margins’ are not even on the horizon of thinking when any design, plan or institutional framework for health delivery is developed. These factors restrict or inhibit LGBT individuals from accessing healthcare [66]. Because of the insensitivity of the health system, LGBT individuals often have to seek unsafe or delayed treatment or even skip treatment [67]. Moreover, they may also choose not to avail free or subsidised public health facilities and instead go to private providers, which could prove exorbitant in terms of cost and in some instances, even unsafe.

Studies in India and elsewhere indicate that the mental health status of the LGBT community is poor and often alarming. Family support plays a major role in mitigating stigma and the resulting mental stress. Married MSM were found to have “*lower rates of depression than unmarried MSM*” individuals [47]. However, being “*forced into different-sex marriages also creates ...stress, especially for lesbians, for whom marriage*” is likely to result in a stricter set of social roles and reduced freedom [47].

While health issues and health inequity for LGBT individuals predominantly result from the stigma and social exclusion stemming from the lack of recognition and their socially “*devalued identities*” [6], there are also health concerns uniquely associated with being LGBT. Risks associated with unregulated sex reassignment surgeries [68], costs of hormones, gender dysphoria, the risk of chronic diseases, sexually transmitted infections, respiratory tract infections and HIV/AIDS are some such concerns [66]. In the United States, relatively higher proportions of trans youth displayed symptoms of eating disorders, probably due to psychological stress and wanting to comply to bodily standards set by heteronormative society [69].

It is, however, crucial to note that the LGBT community is not a homogenous group of individuals. It would be grossly inaccurate and insensitive to classify all of them and their health issues as being the same. For instance, lesbians are particularly exposed to issues such as corrective rape [3]. Another study points out that a pathologising attitude towards lesbians results in severe medical abuses [45], with lesbians being treated with heavy psychopharmacological drugs for what is perceived as a psychological disorder causing their homosexual orientation. Many trans men, for instance, “*retain their ovaries and uterus as well as the capacity to become pregnant*”. However, health systems are hardly sensitive or equipped to deal with their reproductive needs. Moreover, society is far from accepting trans men as being “*openly pregnant and giving birth*” [70].

### HIV/AIDS discourse, LGBT identity and data

While the Indian Census 2011 collected data on trans individuals as ‘others’, other national level health surveys in India are yet to recognise LGBT as a demographic category. Several countries have attempted to collect data on LGBT individuals. For instance, Nepal, like India, included a third gender category to collect basic demographic data in 2011. Brazil and Uruguay, in their most recent censuses, included questions about same-sex partnerships, and more countries have begun to include items based on sexual orientation and gender identity on more large-scale surveys, allowing for a more detailed analysis [71]. It should, however, be noted that, with stigma and fear of discrimination, there is all likelihood of data on sexual orientation and gender identity being underreported. Until recently, data on general health status regarding LGBT almost solely came from HIV/AIDS-related research. HIV/AIDS was the “*primary entry point for LGBT issues*” [45] in global and national levels for health systems and most non-governmental organisations across the world. It gave visibility but in an unfortunate way. HIV/AIDS interventions were targeted at gay men and funding to LGBT organisations were directed towards regulating MSM while it ignored other categories because of the low prevalence of the infection among other groups within the LGBT umbrella. This representation of LGBT with a focus on MSM gave a skewed picture, which became a problem when this discussion was extended to other realms. For instance, lesbians or women who had sex with women but did not identify as lesbians were considered to be less relevant and therefore very little HIV/AIDS related research focused on them [47]. By pathologising and labelling LGBT as high-risk groups, the campaign against HIV/AIDS created an expansive and sustained discourse that ‘othered’ the LGBT community in an enduring manner. The discourse had no understanding of the politics of ‘queer’ and lacked sensitivity to the underlying complexities. It categorised people based solely on their sexual behaviour as a narrow variable and displayed no understanding of inherent, interrelated and underlying aspects of gender identity and sexual orientation. Further, it gave an individual and illness-based focus to the issue, thus “*erasing the social context of identities*” [68], and in the process ignoring self-affirming gay and bisexual men, lesbian, bisexual women, asexual persons, people with intersex variations and trans persons. The individual-orientation ignored collective aspects and other diverse health-related problems in connection with the LGBT communities [3, 68].

For health systems to be sensitive and plan affirmatively for the welfare of the LGBT community, there is a need to collect data along the dimension of sexual orientation and gender identity. At the same time, one has to be careful that the process of data collection is not intrusive and stigmatising. There is also a need to go beyond mere measurements since the commonly used indicators may not accurately demonstrate the problems of LGBT individuals and may fail to capture the difference along the dimensions of sexual orientation and gender identity, contributing to erasure of identities. Instead, there is a need to balance the efforts with a focus on the lived experience of individuals embedded in their social location and empirical contexts.

## Discussion

In the foregoing discussion, two different approaches to address health inequities along the dimensions of disability, mental health, sexual orientation and gender identity emerge. One approach looks at the lacuna in the current public health discourse in addressing the specific health concerns of persons living with disability, those experiencing mental health problems and LGBT individuals; it also examines their underrepresentation in the various arms of the health systems, such as in human resources, and the invisibility of their issues in the training curricula of healthcare professionals and in policies and programmes. This approach calls for working within the current political, economic and social structures without challenging the status quo, and tries to fix unaddressed problems in a patch-work mode.

The second approach, which we would like to emphasise, involves a more organic way of addressing the concerns of these population groups, rather than including them peripherally as after-thoughts. This involves questioning the way health and equity is understood in terms of ‘closing the gaps’ and rearticulating them in light of the specific concerns brought about by these three dimensions, which is about acknowledging and addressing ‘differences’.

Though one is aware of the social mediation of health conditions and health outcomes, health as a body of knowledge continues to be based on the notion of a naturalised persona that is objective, neutral and inert to social arrangements. As discussed in the preceding sections, this manner of conceptualising health is merely a particular instant of articulation of a discursive entity and is inadequate in representing the concerns of health inequities stemming along these dimensions. The forms of vulnerabilities discussed in this article are not solely based on distributional aspects of inequity but also on recognition and identity [6]. Integrating the dimensions of recognition and identity expands our conceptual framework, giving a central role to the experience of differences, disadvantages and stigma, rather than emphasising only quantifiable differentials in health indicators.

Individual subjective experience of stigma represents and forms the basis of collective identities around disability, mental health, gender identity and sexual orientation. Such experience also forms the basis of the differences between identities. The idea of difference emerges as an important concept in this articulation of health, providing a moral-epistemological location to understand health and health inequity. Recognition and representation of these differences then form the basis of an inclusive articulation on health equity [68]. An unhampered environment for persons with a disability, mental illness or those identifying as queer to lead an independent life as full members of the society in their own terms, without being stigmatised and discriminated on the basis of these identities, will then define health equity. However, this is not to undermine or discount the intersectional role of other dimensions such as class, race, ethnicity and caste in influencing the degrees of inequity even within these groups.

The idea of equity rests on ‘avoidable and unfair differentials’. The discussion in the preceding sections prompts us to problematise these elements. What is avoidable or unavoidable appears to be unambiguous within a band of mainstream society. However, the idea of unavoidable and unfair cannot be objectively demarcated in the context of mental health concerns or in problems associated with ageing and disability. It is often a moral call taken by the society based on the prevailing values.

The concept of differentials inevitably involves measurement. For it to be meaningful, it is required that the entities that are compared have to be comparable and that they have to be located on a single scale, within the same meaning-constituting system; the ideas of difference [72] and experience challenge this possibility. The meaning of two diverse experiences has to be derived separately by treating them as part of two different meaning-constituting systems, appreciating and retaining the difference rather than aiming to measure the differentials by placing them within a single system of meanings.

Arriving at differentials entails a belief in a form of that involves breaking a complex whole into its constituent parts. While such an exercise may be essential for the sake of measurement and comparability, it can marginalise experiences that cannot be mechanically broken down into constituent parts but convey complex layers of meanings as a totality. Any understanding of health inequities that excludes such experiences from the analysis is inadequate. What is required is not aspiring to reduce experiences into differentials but treating the experiences as relevant outcomes for analysis of health inequities, retaining their specificities.

## Conclusion

The dimensions of disability, mental illness, sexual orientation and gender identity contribute to health inequities in many ways. They affect health constitutively among the respective populations, both physically and mentally, and also affect health instrumentally through co-morbidities, making individuals susceptible to health problems and affecting health-promoting activities such as exercises or physical access to healthcare. It is also important to acknowledge the intersectional role of these identities in conjunction with other identities such as caste, race and economic status. Stigma and its associated marginalisation constitute another important pathway through which these dimensions configure health inequities, either directly or indirectly. The direct effect takes the form of discrimination at health facilities, refusal of care, insensitivity and lack of representation. This operates at all levels, ranging from individual and household to community, health system and policy levels. Stigma can also affect health inequity indirectly by excluding individuals and community from education, livelihood opportunities and social networks, thereby pushing them to impoverishment, which in turn could affect their health, nutrition and access to healthcare.

The subjective experience of negotiating a stigmatising and discriminating society and health system is a common element across all the three dimensions of vulnerability. The centrality of discriminatory experience in shaping health offers the scope to question the understanding of inequity that is elucidated only in terms of differentials. It compels us to consider experience as a legitimate and relevant outcome to represent and understand health and health inequities. Therefore, health equity will mandate an inclusive social arrangement that celebrates difference.

## Footnotes

1 We understand the limitations of the acronym LGBT in capturing the entire dimension of sexual and gender identities. Our usage of it is in no way meant to ignore the other identities not captured by L-G-B-T.
